# Supplementary material for: Spatio-temporal dynamics of intra-host variability in SARS-CoV-2 genomes
Source: Nucleic Acids Res. 2022 Jan 20;50(3):1551–61. doi: 10.1093/nar/gkab1297 (PMC8860616; doi:10.1093/nar/gkab1297)
Supplement: gkab1297_Supplemental_Files [file gkab1297_supplemental_files.zip › Supplementary Data.pdf]

SUPPLEMENTARY DATA

	Hydrophobic	Polar	Positive	Negative
**Hydrophobic**	120	66	9	10
**Polar**	80	83	58	29
**Positive**	25	28	16	12
**Negative**	53	32	2	5

Supplementary Table S1: Type of amino acid change at Spike iSNV sites in hyper-variable samples. The iSNV associated Spike variants in the hyper-variable samples characterised according to hydrophobic, polar and charged (positive and negative) nature of the residue. The rows indicate the amino acid property of the original residue and the column indicates the property of the modified residue. The number of iSNV associated variants falling in each of the pair categories are listed.

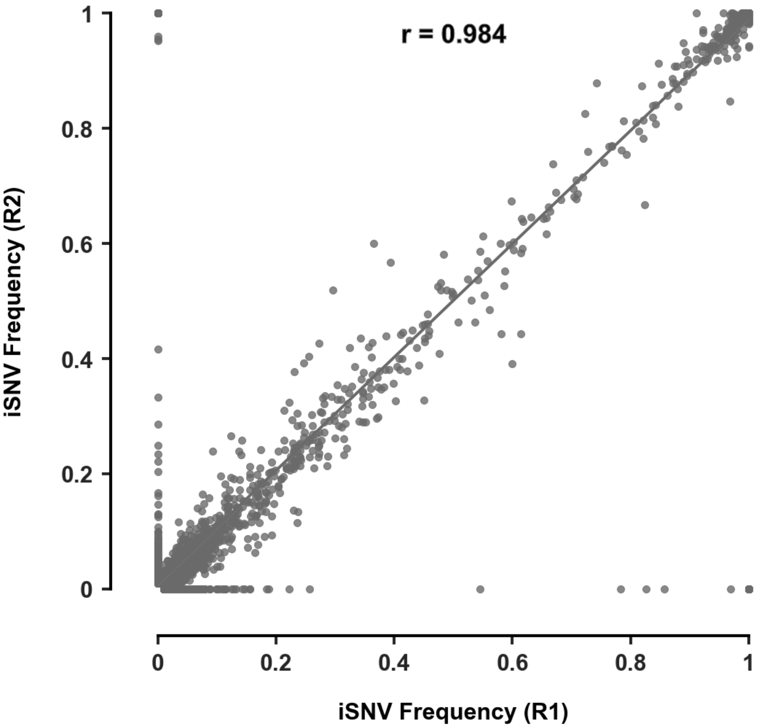

Supplementary Figure S1. iSNV concordance between replicates. Correlation plot illustrating the concordance in iSNV frequencies between replicates (n=500).

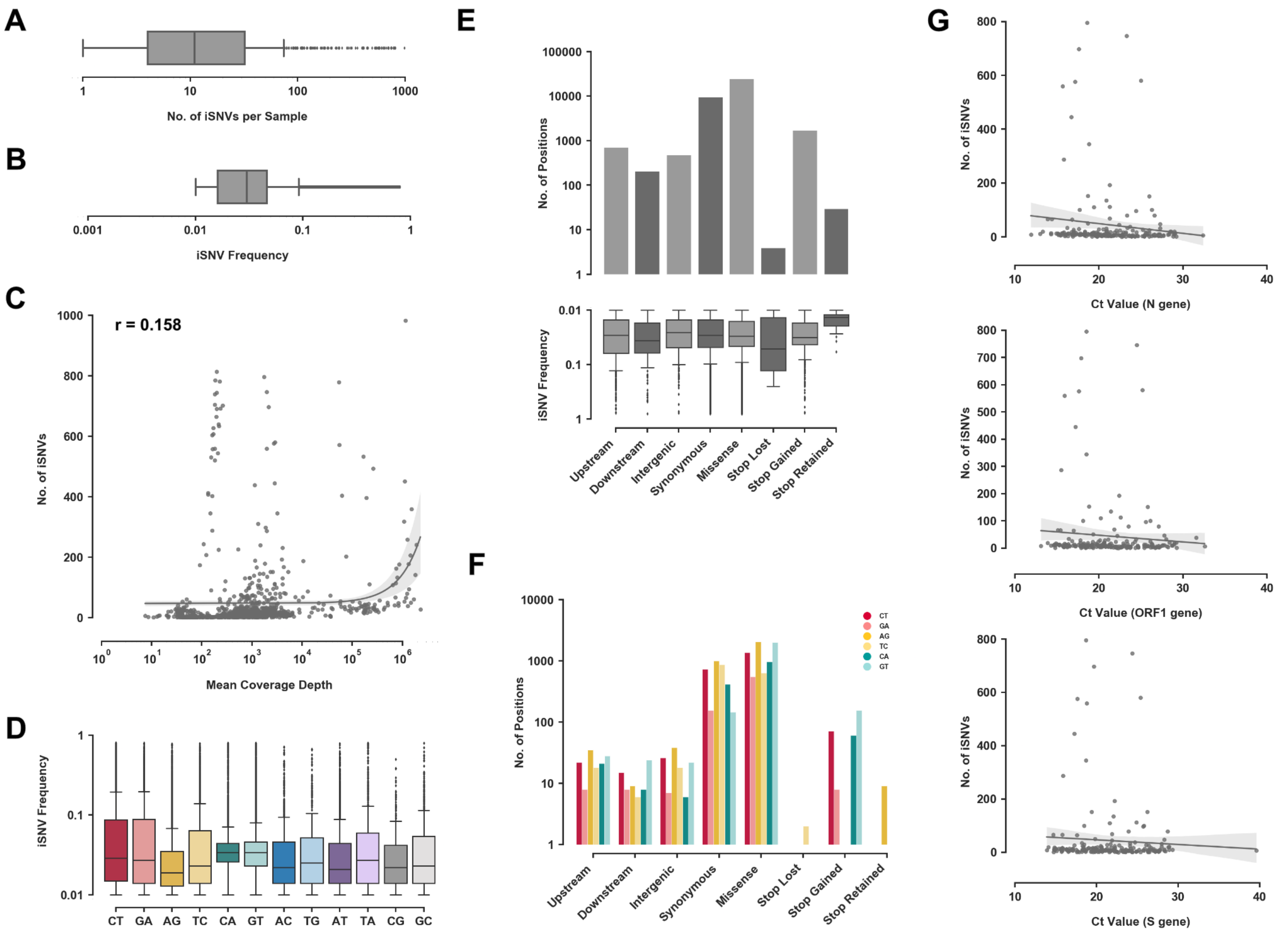

Supplementary Figure S2. Distribution of iSNVs in Phase 1 samples. (**A**) Distribution of the number of iSNVs per sample (n=929) (**B**) Distribution of the frequencies of all captured iSNVs (n=47779) (**C**) Scatter plot to assess the correlation between number of iSNVs and mean coverage depth in samples (n=929) (**D**) Distribution of iSNV frequencies with respect to the nucleotide change (**E**) Split plot showing distribution of number of iSNVs and its potential impact vis-a-vis nature of amino acid substitution (n=37805) (**F**) Distribution of nucleotide change mediated by iSNVs with respect to nature of amino acid sequence change (**G**) Correlation plot to compare the concordance between the number of iSNVs in samples and Ct values of N, ORF1 and S gene respectively in East India cohort.

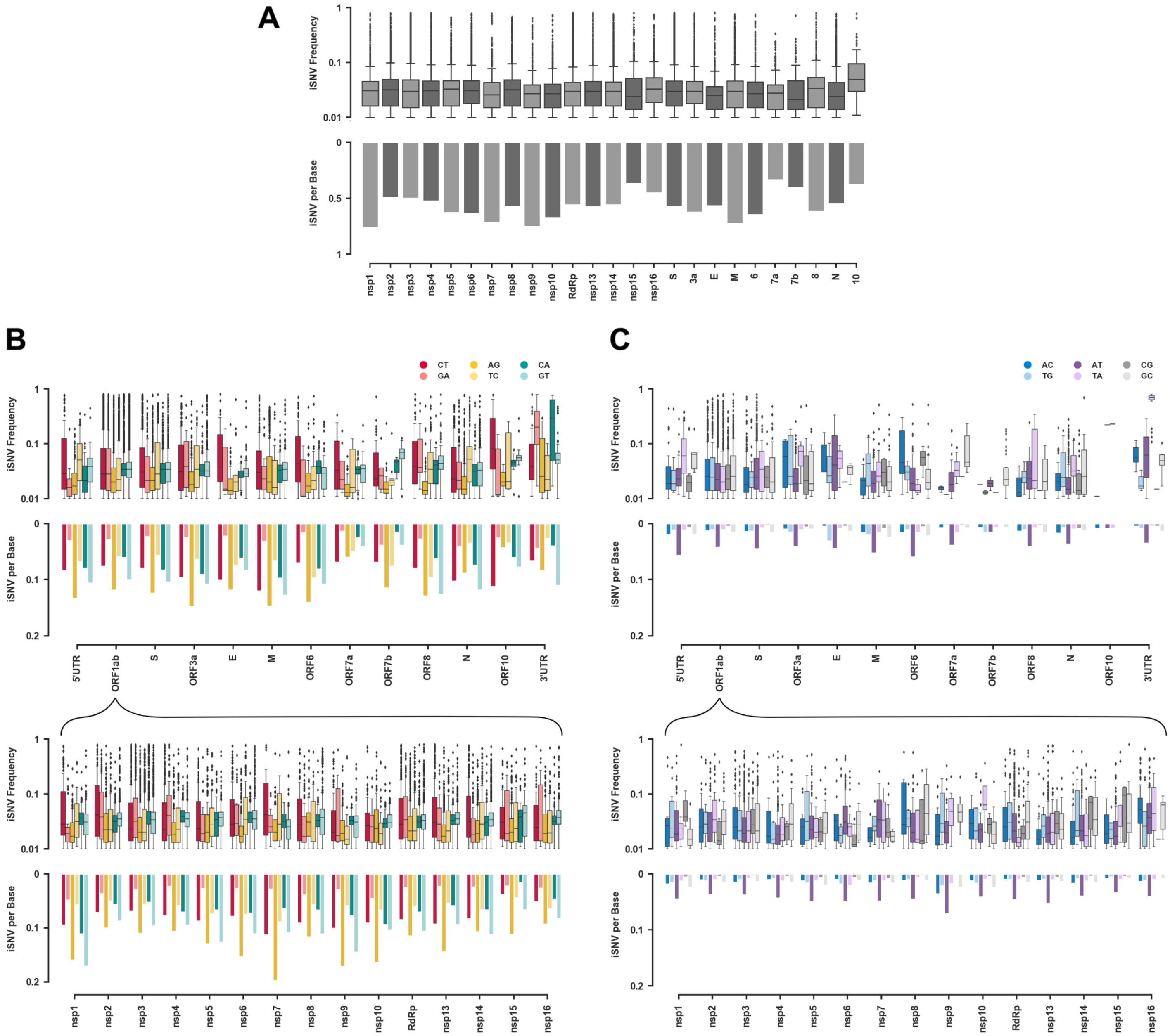

Supplementary Figure S3. Distribution of iSNVs across the SARS-CoV-2 genome. (**A**) Split plot depicting the distribution of iSNV frequencies and number of iSNVs per base in each protein-coding domain. (**B**) Split plot depicting the distribution of iSNV frequencies and number of iSNVs per base in each protein-coding domain with respect to potential APOBEC (CT/GA), ADAR (AG/TC) and ROS (CA/GT) inflicted nucleotide changes. (**C**) Split plot depicting the distribution of iSNV frequencies and number of iSNVs per base in each protein-coding domain with respect to other nucleotide changes.

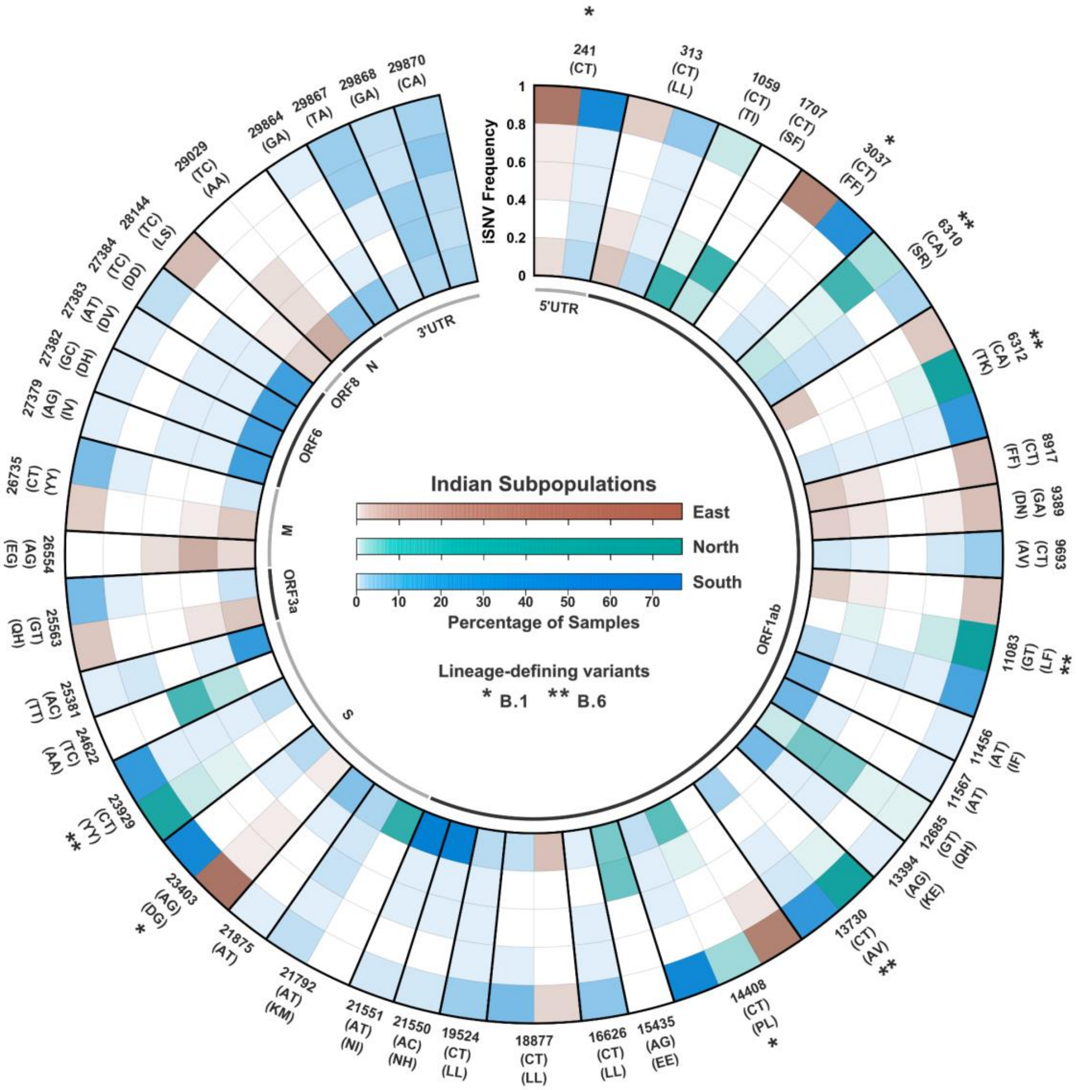

Supplementary Figure S4. Spectrum of iSNVs in Indian subpopulations. Radial plot showcasing frequency distribution for select iSNV sites in East India, North India and South India denoted in Brown, Green and Blue respectively. Each concentric ring depicts an iSNV frequency range of 0.2 and the different colour gradients depict different populations with percentage of samples at a given position in each cell. The outer labels denote the position of change, nucleotide change and amino acid change. Variations that define the B.1 and B.6 lineages have been marked (*) and (**) respectively.

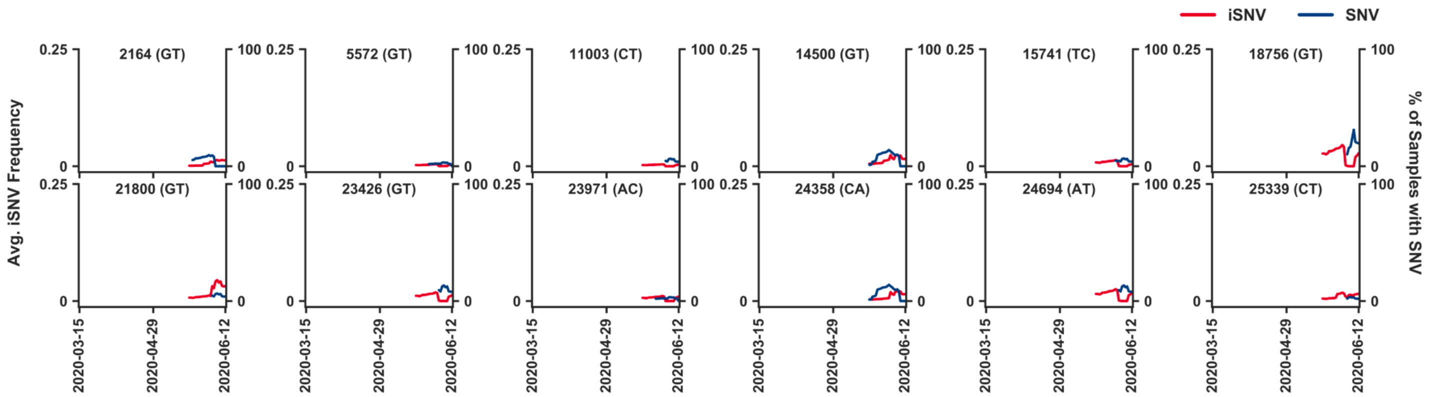

Supplementary Figure S5. Spatio-temporal dynamics of iSNVs in East India. Line plot showing temporal trends of iSNV frequency and incidence of SNV in the East India cohort. The left y-axis denotes the average iSNV frequency and the right y-axis denotes the percentage of samples with SNVs on a 14-day rolling basis. Red and blue lines illustrate the average iSNV frequency and the percentage of samples with SNVs at the site respectively.

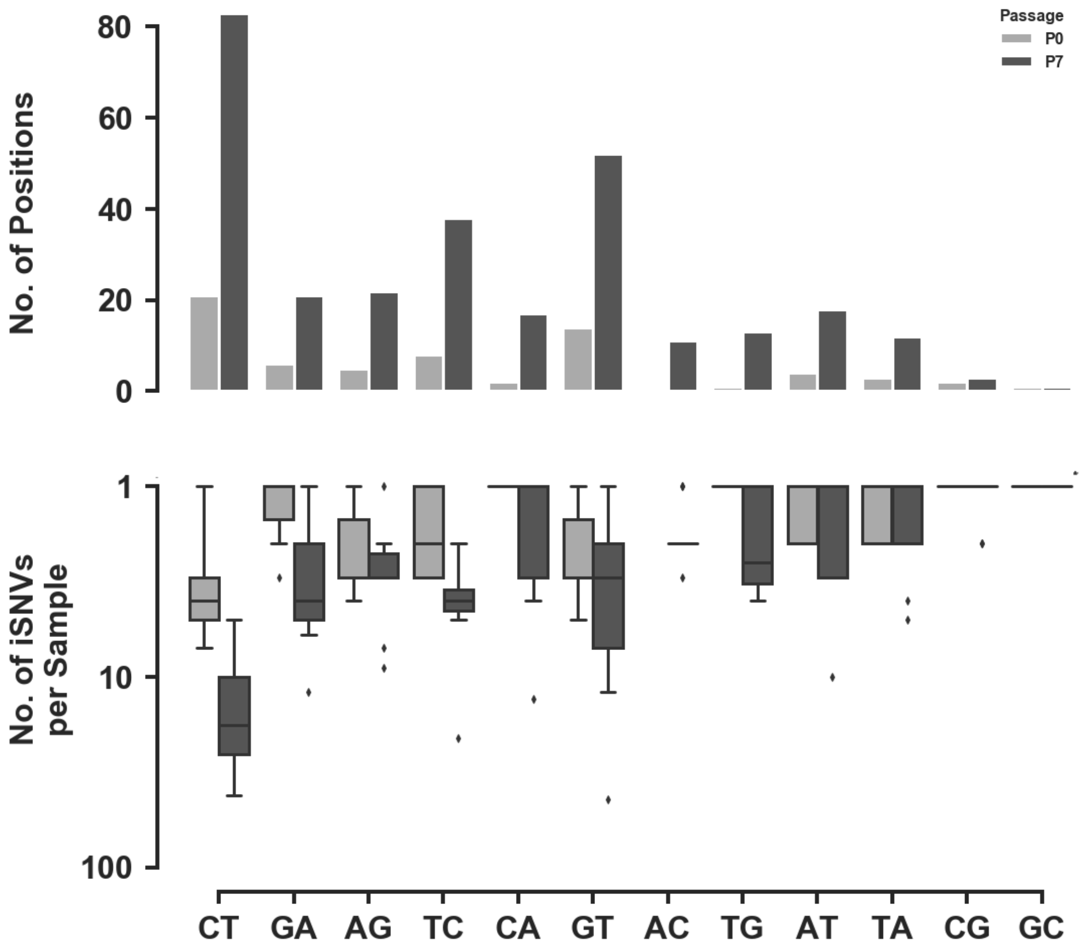

Supplementary Figure S6. Accumulation of iSNVs in Vero cells. Comparison of iSNV prevalence between the original batch (P0) and after 7th passage (P7) of 11 independent isolates of SARS-CoV-2 samples cultured in Vero cells from the East India cohort.

Supplementary Dataset Legends

Supplementary Dataset S1: Coordinates of homopolymeric regions in the SARS-CoV-2 genome

Supplementary Dataset S2: Data summary of pipeline analyses and metadata for each sample

Supplementary Dataset S3a: Phase 1 sample-wise frequencies of all recorded iSNVs in global populations

Supplementary Dataset S3b: Phase 1 sample-wise frequencies of all recorded iSNVs in Indian subpopulations

Supplementary Dataset S4a-d: Phase 2 sample-wise frequencies of all recorded iSNVs

Supplementary Dataset S5: Summary of nucleotide change, codon change and amino acid sequence change of all recorded iSNV sites in Phase 1 samples and the number of samples with respective SNVs in GISAID

Supplementary Dataset S6: Potential C14408T and C13730T mutant RdRp associated iSNVs

Supplementary Dataset S7: qRT-PCR results for mRNA expression of APOBEC3B and ADARB1 genes

Supplementary Dataset S8: Sample-wise frequencies of all recorded iSNVs in SARS-CoV-2 infected cultures of Vero E6 cells

Supplementary Dataset S9: iSNVs mapped to lineage-defining variants of VOCs and VOIs listed by the WHO by 30th June 2021

Supplementary Dataset S10: iSNVs mapped to potential immune-escape variants
